# Treatment of Orchitis

**Published:** 1886-03

**Authors:** A. S. Wolf

**Affiliations:** Brownsville, Texas


### ON THE TREATMENT OF ORCHITIS BY DIVISION OF THE TUNICS OF THE TESTICLE.

*By A. S. Wolf, M.D., Brownsville, Texas.*

For Daniel's Texas Medical Journal.

ORCHITIS as a result of gonorrhœa is so frequent that it is of peculiar interest to the surgeon.

Parenchymatous orchitis scarcely ever exists without epididymitis. This inflammation precedes orchitis. In order to extend itself to the testis, the inflammation must affect the epididymis ;—be it understood that I refer to that affection consequent on gonorrhœa.

In cases following blows on the organ, or wounds of its structure, the reverse may happen ; we may meet with parenchymatous orchitis before we have epididymitis ; we can easily conceive the existence of the first inflammation in such cases, without the coincidence of the second. The tumor, however, formed by the inflamed testis, is less voluminous than when caused by epididymitis.

Parenchymatous orchitis, like epididymitis, recognizes as its cause most frequently, an existing gonorrhœa, or one that has recently disappeared. It is a common observation that when inflammation of the testis supervenes in gonorrhœa, the pain in making water and urethral discharges ceases, or undergoes considerable diminution, but returns as the orchitis subsides.

The testis is not much enlarged, owing to the unyielding nature of the tunica albuginae ; its vessels are congested. The epididymis is enlarged, especially at the lower part, to twice or three times its natural size, and feels thick, firm and indurated. This enlargement depends on the presence of exudation matter. The coats of the *vasa deferens* are thickened and the adjacent vessels injected. The tunica vaginalis is inflamed, and its cavity contains the usual effusion.

Orchitis is characterized by a painful swelling. Inflammation of the testis and epididymis, often involves the spermatic cord, and especially the *vas deferens.*

Gonorrhœa injudiciously suppressed by astringents or saturnine injections into the urethra, may extend by a succession of continuity to the ejaculary canal, *vesicula seminalis*, *vas deferens*, and thence to the *epididymis* ; the spermatic vein often participates in the misfortune by becoming varicosed.

Inflammation of the testis is far more frequently met with as a consecutive affection than as a primary one. The gland is directly connected through the medium of the *vas deferens* with the urinary organs, the lining membrane of its numerous minute ducts being continuous with the mucous membrane of the urethra. Any irritation, therefore, affecting that part of the urethra where the vasa deferentia terminates, is liable to be propagated to the testis, and causes it to inflame. Acute orchitis, being a frequent sequel to gonorrhœa, it may supervene at all periods, during its early and acute stage, as well as during its termination, though it more frequently commences when the pain and discharge begin to subside.

In epididymitis the swelling forms the larger portion next to the testis, and lastly in the tunica vaginalis. The form of the partial tumor referred to is that of the testis—exagerated, it is true.

The inflamed organ presents a swelling in front of the epididymis and behind the serous effusion, the swelling is as if it contained coagulated fluid. When the tunica vaginalis is emptied, the testis appears to occupy the place of the liquid, and this swelling, previously pear-shaped, assumes a round form.

The pain and local heat are of the most violent form. The pain has this peculiarity, that it spread upwards and downwards, extending to the kidneys, and corresponding points below. The color of the skin is generally slightly modified, but in some cases it is exceedingly red, or even violet color.

It has been remarked that the cord is less liable to be affected in cases of parenchymatous orchitis than in instances of simple epididymitis.

Fever is generally present; there is sleeplessness, nausea, colic, and vomiting comes on, adding considerably to the suffering of the patient.

We all know what a terrible ordeal the sufferer must undergo in inflammation of the testicle. After laying in bed for several days, constant application of ice, or the converse (warm applications may be resorted to), the unfortunate wretch has to be purged, then evaporating lotions to be applied, and what not? Then, when matters begin to mend, the misery which awaits him, the prolonged torture he has to undergo when the organ is left to the attendant, be he ever so skillful in strapping.

Fourneau Jourdan advises painting the scrotum with arg. nit., while a stripe of vesication is exited over the adjacent femoral artery.

Noble Smith, of St. Mary's Hospital, advises caustic and evaporating lotions ; and many others have wrung the changes on different treatments with more or less success.

If we consider the ill effects and the evil consequence that may follow, to one who is suffering from orchitis, the danger to which he is liable, even to the impairment of the organ, leading, may be, to impotence, anything, or any one who will suggest a plan of treatment which promises to shorten the suffering and danger to one suffering from orchitis, old as it may be, should receive due consideration.

The prognosis is not severe when we loose no time in destroying that which gives it particular character, namely, strangulation.

This was successfully accomplished by my countryman, and, as far as I know, for the first time, Dr. Vidal de Casis, who recommends incision, and reports four hundred cases without untoward accident. This mode of procedure found favor with Dr. Cullelier, of Paris, Dr. Mum, of Middlesex Hospital, London, and Dr. Smith, of King's College Hospital, London.

It consists of making an incision of 0.01 centimetre, layer by layer down to the albuginæ; there being no serosity within the tunica vaginalis, union without suppuration takes place between the testis and vaginalis at the incision. Should there be some serous infiltration in the latter, a small incision, without necessitating division of all the layers down to the albuginæ, may be sufficient to afford immediate relief. The pain ceases, the fever decreases, and the patient soon falls asleep, although he has been unable to rest for days.

There is nothing alarming to the patient, in the little operation; it should be classed with iridectomy in acute glaucoma, an operation essentially done for the relief of tension, the fibrous sclerotic of the eye, and the tunica albuginæ testis having at least a family likeness.

No further application is necessary other than a laudanum poultice applied to the scrotum. The patient confined to his bed, with the testis raised on a small pillow between the thighs.

For fear of being accused of diffuseness, I spare the space of your valuable journal and the reader the enumeration of some sixty cases, during a long practice, treated by incision as recommended by Dr. Vidal de Casis. The following two cases will,

I hope, be sufficient to prove the great value of the old practice, all the other cases offering about the same characteristics.

J. H., a carpenter, 28 years of age, a strong and healthy young fellow, came under my care under the following circumstances: He contracted gonorrhœa about three weeks before he had received treatment from a druggist; the last was an injection zinci. sulph. The discharge was nearly stopped; the testicle, however, became swollen and painful. He kept at work, but on the third day the organ gave him so much pain that he became alarmed, and applied to me. I found him in bed suffering great agony, the testicle enormously swollen, the scrotum violet in color. The acute stage had not yet passed off. Without waiting for the acute symptoms to subside, I made an incision as practiced by Dr. de Casis. A few hours after he went to sleep, and that was the last of the case, having had no further trouble with him.

W. H., age 19; twice gonorrhœa before. For the last and third one he took the same medicine as that which was prescribed for him by a regular physician on previous occasions. The discharge was nearly gone, and, pleased with the success of his own doctoring, went about his usual avocation, necessitating much exercise. After two days, the testicle began to hurt him, and soon the pain was so acute that he went home, and sent for me. I found him with a great deal of constitutional disturbance, he had not slept for two days, the discharge was entirely stopped. I immediately made an incision through the dartos and through the tunica vaginalis. There was some exudation of fluid, which made its escape at the incision. I applied a laudanum poultice, and that was the last of that case.

Some ten days after he called at my office. The discharge of the urethra had re-appeared, which which soon yielded by appropriate treatment.

I am free to state, that in all my cases, nothing has supervened to make me lose faith and confidence in incision for orchitis. Of course there is nothing new or startling in the practice. All are familiar with the division of the fibrous tissue of the testicle; but may I not ask, without being impertinent, how many practice it?

I advance no new theory, extoll no new remedy. There is nothing original in the foregoing. I merely wish to call attention of my fellow practitioners to a treatment, old as it may be, that possesses unusual merits, in cases of parenchymatous orchitis.

Still further, I believe it is of some importance to record our individual experience, above all, referring to subjects on which there is paucity in our literature. Few, if any, of the systematic writers make reference to Dr. Vidal de Casis' procedure and treatment. It is as a reminder that this paper suggested itself to me.

Besides, there is not a fact nor an observation, nay, scarce a book, or an article so badly furnished in the medical literature, from which some good may not be gathered, if sought for in the right and kindly spirit. Among the veriest dross some good may linger. From these littles a magnificent whole may one day be formed. There is not one fact too insignificant for preservation, till all, united by some master mind, some peculiar genius, who, in the summing up of all, creates a whole.

